# To Live or to Die: Prosurvival Activity of PPAR**γ** in Cancers

**DOI:** 10.1155/2008/209629

**Published:** 2008-09-08

**Authors:** Y. Lynn Wang, Qi Miao

**Affiliations:** Molecular Hematopathology Laboratory, Department of Pathology and Laboratory Medicine, Weill Cornell Medical College, New York, NY 10065, USA

## Abstract

The role of PPAR*γ* in tumorigenesis is controversial. In this article, we review and analyze literature from the past decade that highlights the potential proneoplastic activity of PPAR*γ*. We discuss the following five aspects of the nuclear hormone receptor and its agonists: (1) relative expression of PPAR*γ* in human tumor *versus* normal tissues; (2) receptor-dependent proneoplastic effects; (3) impact of PPAR*γ* and its agonists on tumors in animal models; (4) clinical trials of thiazolidinediones (TZDs) in human malignancies; (5) TZDs as chemopreventive agents in epidemiology studies. The focus is placed on the most relevant *in vivo* animal models and human data. *In vitro* cell line studies are included only when the effects are shown to be dependent on the PPAR*γ* receptor.

## 1. INTRODUCTION

PPAR*γ* is a nuclear hormone receptor that requires ligand binding for activation. In 1995, it was discovered that PPAR*γ* is the molecular target of thiazolidinediones (TZDs, [1]), a class of synthetic compounds that are effective for the treatment of type 2 diabetes. This discovery spurred great interest in these agents, as well as in the receptor. Besides its function as an insulin sensitizer in diabetes, PPAR*γ* was found to have a variety of roles in immunoregulation, atherosclerosis, angiogenesis, and tumorigenesis.

With regards to carcinogenesis, debate continues as to whether PPAR*γ* is pro- or antineoplastic, despite very active research over the past few years. At the cellular level, PPAR*γ* was found to be involved in cancer cell survival/apoptosis, proliferation, and differentiation. While the apoptotic functions of PPAR*γ* and its agonists are addressed by others in this special issue, we will conduct a critical review of the literature that suggests that PPAR*γ* has a prosurvival activity. The review is mainly focused on data derived from *in vivo* models and/or human studies. *In vitro* cell line-based studies are included only when the effects are shown to be dependent on the PPAR*γ* receptor.

One important lesson learned from the past several years of research is that effects observed with agonists of PPAR*γ* are not necessarily intrinsic effects of the nuclear hormone receptor. In tumor cell survival, the proapoptotic activities of PPAR*γ* agonists in various tumors act through both receptor-dependent and receptor-independent mechanisms. When reviewing the literature, we advise that the readers carefully consider the following to distinguish drugs or TZDs *versus* receptor effects: (1) are high or low doses used in the studies? High or low doses should be defined with respect to EC_50_ of glitazones in the PPAR*γ* transactivation assays (Table 1) or plasma concentrations that can be reached in humans (Table 2). Effects observed with high concentrations may not be relevant due to toxicities of certain TZDs, such as hepatotoxicity of troglitazone and potential cardiotoxicity of rosiglitazone (see below). (2) Are multiple pharmacological agents used? If a pharmacological approach is the only one used, claims of a receptor-dependent effect require demonstration with agonists of different chemical structures, such as TZDs, tyrosine analogues, 15-Deoxy-Δ^12,14^-PGJ_2_ (15d-PGJ_2_), and so forth. Beware that 15d-PGJ_2_ possesses many PPAR*γ*-independent activities, including inhibition of the NF*κ*B pathway, that are known to have prosurvival and anti-inflammatory properties, as well as other effects [2–4]. (3) Are any antagonists included in the study? Do antagonists GW9662 or T0070907 block or reverse the observed effects? (4) Are there any experiments in the study utilizing a genetic approach to confirm the pharmacological findings? Does the study involve cell lines or primary cells that contain or lack PPAR*γ*, preferably in the same genetic background? For those cell lines with endogenous PPAR*γ*, is the siRNA, shRNA or dominant negative form of PPAR*γ* used to reduce the levels of the receptor? Are specific effects of the receptor diminished by such reduction? For readers' convenience, these questions are summarized in Table 3.

## 2. EXPRESSION OF PPAR*γ* IN HUMAN TUMOR VERSUS NORMAL TISSUES

It is generally believed that expression of a gene in a particular tissue suggests that the activity of the encoded protein is required for certain cellular functions of that tissue. In so far as cancers are concerned, the general rule is that oncogenes are overexpressed due to dysregulation, and tumor suppressor genes are underexpressed or absent due to mutations or deletions. In order to clarify the roles of the PPAR*γ* receptor, it would be informative to review the expression levels of PPAR*γ* in tumors with respect to their normal tissue counterparts. In this article, expression data from tumor cell lines are not included.

A review of the current literature on human cancers showed that expression levels of PPAR*γ* mRNA and protein are generally higher in neoplastic tissues than their normal counterparts (summarized in Table 4). The most convincing data came from a large study of prostate cancer that included 156 patients with prostate cancer (PC), 15 with less aggressive prostatic intraepithelial neoplasia (PIN), 20 with benign prostatic hyperplasia, and 12 normal prostate tissues. In this study, a high level of PPAR*γ* expression, by immunohistochemistry, is observed in PC and PIN cases in comparison to low or no expression in the benign hyperplasia and normal tissues. The results were confirmed at the mRNA level with RT-PCR on a few cases from each category of the malignant and benign conditions [13]. A large study of 126 renal cell carcinomas also showed significantly more extensive and intensive PPAR*γ* staining in tumor epithelium compared to the average staining levels seen in 20 normal tissues [14]. Similarly, in 22 patients with nonsmall cell lung carcinoma, higher levels of PPAR*γ* are expressed in tumor cells than in the surrounding normal tissue, as determined by immunohistochemical staining. In addition, higher expression levels in tumor cells are confirmed by Western blotting hybridization, using homogenized tissue samples [15]. In hepatocellular carcinoma, immunostaining also demonstrates that PPAR*γ* is overexpressed in all of 20 carcinoma tissues but not in normal hepatocytes [16]. For squamous cell carcinoma, 20 cases of primary tumor and six cases of lymph node metastasis were demonstrated to have increased PPAR*γ* protein expression compared to normal tongue tissue [17]. Infiltrating adenocarcinoma of the breast also expresses higher nuclear staining of PPAR*γ* compared to normal ductal epithelial cells by immunohistochemical analysis. However, only one of the three cases was shown [18]. For papillary thyroid carcinoma, six patients were studied to determine PPAR*γ* mRNA expression using reverse transcription PCR. The message was found in three of six tumor tissues while the corresponding normal tissues do not express PPAR*γ* [19].

Follicular thyroid carcinoma, a less common histological subtype of thyroid cancer, is characterized by a chromosomal translocation t(2;3) that results in a fusion between paired box gene 8 on chromosome 2 and PPAR*γ* on chromosome 3 (PAX8-PPAR*γ*). The fusion protein was initially thought to function as a dominant-negative inhibitor of the wild-type PPAR*γ* protein [28]. However, a recent microarray study revealed that (1) PPAR*γ* transcript levels in all seven cases of PAX8-PPAR*γ*-containing follicular carcinomas are more than 10-fold higher than normal thyroid tissues, as determined by both microarray and quantitative RT-PCR analyses; (2) the expression profile of the fusion-positive follicular carcinomas shows induction of genes that are involved in fatty acid, amino acid, and glucose metabolic pathways. Interestingly, many of the upregulated genes are known transcriptional targets of the wild-type receptor, suggesting that the PAX8-PPAR*γ* fusion protein functions similarly to wild-type PPAR*γ*, rather than antagonizing its activity. (3) Using cell lines transfected with PPAR*γ* or the fusion protein, it is shown that expression of some genes, including angiogenic factors PGF and ANGPTL4, is specifically upregulated by the fusion protein, particularly in the absence of ligand, indicating that the fusion protein is constitutively active. Taken together, these experimental data suggest that the translocation enhances the function of PPAR*γ* in a way that contributes to the development or progression of follicular carcinoma of the thyroid [29].

Upregulation of PPAR*γ* has been demonstrated during tumor progression. Mueller et al. have found significant PPAR*γ* staining in six cases of metastatic breast adenocarcinoma. In cell lines established from the primary and metastatic tumors of one of these patients, significantly higher amounts of PPAR*γ* transcript are shown in the cell line derived from the metastatic tumor [20]. In ovarian cancer, intensity and location of PPAR*γ* immunostaining were examined in 28 carcinoma cases along with 28 normal, benign or borderline cases. Twenty six of 28 carcinomas showed strongly positive PPAR*γ* staining compared to 2 weak-staining cases in the control group. Moreover, it is noted that PPAR*γ* staining was predominantly nuclear in grade 2 or 3 tumors, as compared to a predominantly cytoplasmic staining pattern in grade 1 tumors [21]. Similar findings were made in transitional cell carcinoma of urinary bladder. Whereas no significant PPAR*γ* immunoreactivity was observed in 20 normal tissues, elevated PPAR*γ* was found in 168 tumors. Furthermore, the intensity of staining increased as the histological grade increased from G1 to G3 and the tumor stage increased from early (pT1 or lower) to advanced (stage 2 or higher) [22].

A recent large study of 129 cases of pancreatic ductal adenocarcinoma convincingly showed by array-based gene profiling that expression of PPAR*γ* in the tumor cells is ~7 fold higher than that in the normal ductal epithelia. This finding was confirmed with immunohistochemical analysis of the tissue sections. Normal ductal epithelia showed insignificant staining for PPAR*γ*. An early lesion, intraepithelial neoplasia showed occasional PPAR*γ* expression whereas more than 70% of invasive pancreatic carcinoma demonstrated weak to strong expression. Statistical analysis indeed revealed that expression of PPAR*γ* correlates with high tumor stage and higher tumor histological grade. More strikingly, expression of PPAR*γ* in pancreatic cancer is shown, by multivariant survival analysis, to be a significant prognostic indicator for shortened patient survival [23].

In parallel to the above literature, levels of PPAR*γ* mRNA found in several well- or poorly-differentiated colorectal adenocarcinomas, were similar to normal tissues [24]. Another group also found that the PPAR*γ* immunostaining in well-, moderately-, or poorly-differentiated gastric adenocarcinomas is comparable to that in noncancerous tissue adjacent to the tumor [25]. In liposarcomas, PPAR*γ* transcript levels are similar to that of the adipose tissue [26]. In adrenal glands, there is, again, no significant difference in mRNA expression among cases of carcinoma, adenoma, and normal tissues [27]. Notably, at the time of composition of this manuscript, we have not yet found any reports stating that PPAR*γ* expression is downregulated or absent in human tumor *versus* normal tissues (Table 4).

The next question is whether or not the PPAR*γ* expressed in tumor tissues is functional. Are ligands of PPAR*γ* present in the tumor tissues? A thorough and up to date literature search yielded few results. The English abstract of a study published in a foreign language stated that there was no significant difference in 15d-PGJ_2_ concentration between gastric cancer tissues and controls [30]. An earlier study showed that 15d-PGJ_2_ promotes the proliferation of HCA-7, a cyclooxygenase 2 (COX-2)-containing colon cancer cell line at nanomolar concentrations. Further characterization by HPLC and mass spectrometry identified PGJ_2_, a chemical precursor of 15d-PGJ_2_ in the culture medium of HCA-7 cells [31]. COX-2 is a key enzyme in the biochemical pathway that leads to the formation of cyclopentenone prostaglandins including 15d-PGJ_2_. Overexpression of COX-2 has been documented in many cancer types and contributes to tumor growth [32]. Overall, these few and somewhat circumstantial evidences suggest that 15d-PGJ_2_ might be present in the tumor tissues.

Does PPAR*γ* lose or gain abnormal functions through mutations other than PAX8-PPAR*γ* translocation? A large survey of human tumor samples and cancer cell lines does not support such a notion. The exon 3 and 5 mutations, once reported in sporadic colon cancers [33], were not present in nearly 400 cell lines and primary tumor samples including lung, breast, prostate, colon cancers, and leukemias [34].

Taken together, several lines of evidence regarding PPAR*γ* expression suggest a positive contributive role of the receptor in the development, maintenance, or progression of human malignancies: (1) PPAR*γ* is overexpressed in the vast majority of cancers. (2) In several types of cancer, PPAR*γ* expression is further increased during tumor progression. (3) The oncogenic fusion PAX8-PPAR*γ* results in PPAR*γ* overexpression and upregulation of a similar profile of transcriptional targets as the wild-type protein. (4) Expression of PPAR*γ* in pancreatic cancer is associated with shorter survival.

## 3. RECEPTOR-DEPENDENT PRONEOPLASTIC EFFECTS OF PPAR*γ*

Is there also cellular-level evidence suggesting that PPAR*γ* promotes tumors? Most studies, especially those employing high doses of TZDs, suggest that PPAR*γ* agonists have antitumor activities through inhibition of cell proliferation or induction of apoptosis or differentiation. However, receptor-independent pathways are involved in most of the cases (reviewed elsewhere in this special issue). Then what does the receptor by itself do in tumors?

Schaefer et al. showed that inhibition of PPAR*γ* induces apoptosis of hepatocellular carcinoma cells (HCCs) by preventing their adhesion to the extracellular matrix, suggesting that the activity of PPAR*γ* is required for HCC cells to adhere and survive [16]. In that study, those particular effects were shown to be receptor-dependent. Loss of cell adhesion requires almost complete loss of PPAR*γ* activity achieved by either PPAR*γ*-targeting siRNA or PPAR*γ* inhibitor T0070907. In addition, T0070907 causes cell death at concentrations far lower than those needed for PPAR*γ* agonists rosiglitazone and troglitazone. Together, the data suggest that PPAR*γ* functions to promote tumor cell adhesion and survival in HCC cells. In line with this notion, the promoter region of hepatocyte growth factor contains a functional PPAR response element (PPRE) that mediates its transcriptional upregulation by PPAR*γ*. The growth factor plays an essential role in liver growth during embryonic development, as well as in maintenance and renewal of cells in various organs including liver, lung, and kidney, in adulthood [35].

Our laboratory studied human anaplastic large T-cell lymphomas, a common form of large cell lymphoma in the pediatric population. We first demonstrated with immunohistochemical staining that PPAR*γ* is expressed in the malignant cells of the lymphoma tissues [36]. We then tested the effect of PPAR*γ* activation in cell lines established from patients with this lymphoma. A pair of cell lines, Karpas 299 and SUP-M2 that, respectively, contain and lack endogenous PPAR*γ* were selected to address the receptor-dependency issue. Additionally, only low ligand concentrations were used, following initial dose titration, to minimize any off-target effects. Using this system, we have found that low doses of PPAR*γ* agonists do not affect cell survival under normal conditions. When cell death was induced by nutrient deprivation through serum withdrawal, activation of the receptor with low doses of rosiglitazone (0.5–2 *μ*M) attenuated cell death, as compared to drug vehicle-treated cells. This result was reproducible with low doses of GW7845 (0.5–2 *μ*M) and 15d-PGJ_2_(0.5–1 *μ*M). The effect occurred only in PPAR*γ*-containing Karpas 299 cells but not in PPAR*γ*-lacking SUP-M2 cells. Moreover, reducing PPAR*γ* in Karpas 299 cells with siRNA diminished the prosurvival effect of the receptor. Furthermore, we showed that the prosurvival effect is mediated through PPAR*γ*-dependent cellular metabolic changes, including increased cellular ATP levels, stabilized mitochondrial membrane potential, and reduced reactive oxygen species (ROS) production that each favor cell survival. PPAR*γ* does so through coordinated regulation of the expression of ROS metabolic enzymes, including the p67 subunit of NADPH oxidase, uncoupling protein 2 (UCP2), and manganese superoxide dismutase (Mn-SOD) at both mRNA and protein levels that lead to ROS limitation. Lastly, we showed that stable transfection of PPAR*γ* into SUP-M2 cells not only improved cell survival, but also suppressed ROS accumulation during serum starvation. These genetic manipulations have provided definitive evidence that PPAR*γ* promotes lymphoma cell survival under conditions of nutrient deprivation.

**Figure 1:**
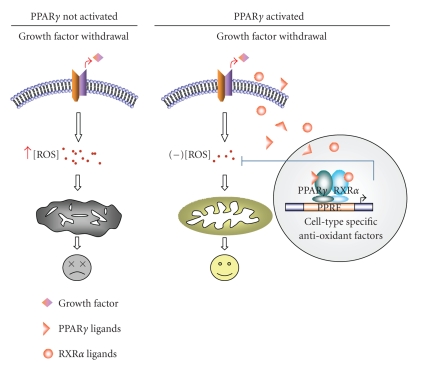
*Schematic diagram showing how PPAR*γ* increases cell survival in growth factor/nutrient-deprived cells*. Growth factor/nutrient withdrawal induces ROS production. In the absence of PPAR*γ* activation, increased levels of ROS inhibit mitochondrial electron transport, leading to mitochondrial depolarization, caspase activation, and cell death. When PPAR*γ* is activated, the increase in ROS is attenuated by the receptor through transcriptional upregulation of cell type specific antioxidant factors, such as catalase, Cu/Zn-SOD (SOD1), Mn-SOD (SOD2), or UCP2. The transcriptional upregulation of these genes by PPAR*γ* may or may not be direct (shown to be direct in the diagram for simplicity).

Our group has also made similar findings in a murine cellular model [37, 38]. FL5.12 is a murine lymphocytic cell line that requires interleukin-3 (IL-3) for survival and proliferation. This cell line has been extensively used to characterize tumor cell metabolism [39]. FL5.12 cells express little PPAR*γ*, but are killed by high concentrations of PPAR*γ* agonists, 15d-PGJ_2_ (≥10 *μ*M) and ciglitazone (≥80 *μ*M). In an FL5.12 cell line stably-transfected with PPAR*γ*, low doses of PPAR*γ* agonist do not affect cell viability under normal conditions. However, when cells are induced to die by IL-3 withdrawal, low doses of ciglitazone (10 *μ*M) and rosiglitazone (0.05–2 *μ*M) improved survival in only PPAR*γ*-containing cells. Improved cell survival is also accompanied by stabilized mitochondria and reduced ROS. Moreover, ATP production is required for PPAR*γ* to exert its prosurvival effect. In this system, expression of a different panel of ROS metabolic enzymes including catalase, and Cu/Zn-SOD are involved in reduction of the cellular levels of ROS. Functional PPRE sequences were shown to be present in the promoter regions of these two genes, suggesting that the upregulation of their expression could be directly regulated by PPAR*γ* [40–42]. Taken together, data from both human and murine cell line studies suggest that PPAR*γ* promotes tumor cell survival under conditions of nutrient/growth factor deprivation, and that the effect is not limited to a particular system. The mechanism by which PPAR*γ* increases cell survival is diagrammed in Figure 1 (Also see below).

In support of the prosurvival activity of PPAR*γ* in T-cell malignancies, Ferreira-Silva et al. very recently showed that RNAi-mediated silencing of PPAR*γ* in Jurkat T-cells caused increased DNA fragmentation and apoptosis as well as G2/M cell cycle arrest, arguing that the receptor, proper, promotes the viability of the tumor cells [43].

In parallel to these findings in tumors, the prosurvival activity of PPAR*γ* has been well documented in certain nonneoplastic pathological conditions, especially ischemia-reperfusion injury in nutrient-sensitive tissues such as brain, heart and kidney [44–51]. Irreversible damage that results from prolonged ischemia causes stroke, and myocardial and kidney infarction. At the cellular level, cell death occurs as a result of nutrient deprivation and inflammatory responses that involve the actions of proinflammatory cytokines, chemokines and transcriptional factors. In addition, increased production of ROS plays an important role in causing damage to macromolecules and eventual cell death [52]. A recent study using a rat model of cerebral focal ischemia has shown that expression of PPAR*γ* mRNA and protein is upregulated in the areas adjacent to infarct caused by middle cerebral artery occlusion [46]. Administration of glitazones prior to, at the time of, or shortly after ischemia induction causes an increase in DNA binding of the receptor. This is accompanied by a decrease in the expression of a number of inflammatory genes, along with an increase in the expression of antioxidant enzymes including catalase and Cu/Zn-SOD [44–47]. Consequently, these changes lead to limited cell demise, which eventually results in significantly reduced infarct size. This process apparently works through a PPAR*γ*-dependent mechanism, as GW9662 can block these effects of TZDs in animals [47]. Another PPAR*γ* antagonist, T0070907, even increases the infarction size, both in the presence and absence of PPAR*γ* ligands [46].

In light of both these findings and the overexpression of PPAR*γ* in many cancers, it is reasonable to hypothesize that the function of PPAR*γ* in cancer is to confer a survival advantage upon the malignant cells, allowing them to survive in an adverse environment. As a result of fast growth, the center of a three dimensional tumor mass is often deprived of oxygen, growth factors, glucose, and other nutrients due to excessive demand and insufficient vascularization. However, cancer cells possess remarkable tolerance and are able to survive despite the adverse conditions [53, 54]. Besides increasing angiogenesis, increasing PPAR*γ* might be another mechanism that allows tumor cells to enhance their survival under these unfavorable conditions (Figure 1).

## 4. IMPACT OF PPAR*γ* AND ITS AGONISTS ON ANIMAL TUMOR MODELS

Animal models were employed to examine the role of PPAR*γ* in tumors. These systems can be categorized by how the tumor models are generated and by how the dose/activity of PPAR*γ* is altered. With respect to the former, tumors can be generated with xenografts, carcinogens, or genetic manipulations. Watch for spontaneous tumor formation in certain PPAR*γ* genetic backgrounds has also been conducted. With respect to the dose/activity of PPAR*γ*, it can be altered using PPAR*γ* agonists including TZDs or GW7845, or genetic manipulations including hemizygosity or tissue-specific overexpression or deletion of PPAR*γ*. Results differ drastically between different model systems, even for the same types of cancer (Tables 5 and 6). This review focuses on models that are more relevant to human cancers. As such, animal studies involving TZD treatment of xenografted tumors are not discussed here.

### 4.1. Colon cancer

*Apc^+/Min^* mice possess a nonsense mutation in one copy of the adenomatous polyposis coli *(APC)* gene which truncates the protein at amino acid 850. Loss-of-function mutations in the *APC* gene are common in human familial adenomatous polyposis and can be found in sporadic colon cancers as well. Using this model, which is highly relevant to human colon cancers, one study showed an increase in tumor number and size, as well as worse histological grade in mice treated with troglitazone or rosiglitazone. This is associated with a rosiglitazone-induced increase in the *β*-catenin protein level in the colon tissues [55]. Another study [56], which also used *Apc^+/Min^* mice, reported an increase in the number of colon polyps in troglitazone-treated mice, but reported no significant difference in tumor size or histology, which may be related to the shorter TZD treatments used in this study (5 weeks as compared to 8 weeks in the first study). Similar findings were made in *Apc* ^+/1638N^:*Mlh1* ^+/−^ double mutant mice. In these mice, one copy of the APC gene is truncated at amino acid position 1638 and one of the two alleles of the DNA repair enzyme *Mlh1* is absent. In the double mutant mice, troglitazone treatment significantly increased the number of mice that developed large intestine tumors [58]. In contrast to these reports, another study used *Apc* ^+/1638N^ mice crossed with hemizygous PPAR*γ* mice. Because homozygous deletion of PPAR*γ* is embryonic-lethal, studies examining the dose effect of the gene employed either a hemizygous *Ppar*γ*^+/−^* mouse strain or a conditional knock-out strategy. No differences in survival, number of colonic tumors or *β*-catenin expression levels were observed between mice of *Apc* ^+/1638N^ :*Ppar* *γ* ^+/−^ and *Apc* ^+/1638N^ :*Ppar* *γ* ^+/+^ littermates [57]. Therefore, in colon cancer induced by *APC* mutations, it appears that activation of PPAR*γ* by TZDs promotes tumor formation, while reduction of PPAR*γ* gene dosage has little effect on tumor formation.

In stark contrast to the *APC* genetic tumor models, carcinogen-generated colon cancer models seem to yield opposite results. In the study that evaluated PPAR*γ* haploinsufficiency in an *Apc* ^+/1638N^ background, the investigators also determined the effect of *Ppar*γ*^+/−^* in azoxymethane-mediated colon cancer. Compared to the *Ppar*γ*^+/+^* mice, a greater number of haploinsufficient mice developed tumors in the colon. The tumor-bearing *Ppar*γ*^+/−^* mice also had a greater number of tumors in them that led to significantly decreased survival. In another study, mice with azoxymethane-mediated colon cancer were treated with troglitazone, pioglitazone, or rosiglitazone. This resulted in reduced incidence, number, and size of colorectal tumor [59]. Taken together, these data suggest that PPAR*γ* suppress azoxymethane-induced colon carcinogenesis.

What would happen in normal mice? Spontaneous colon tumor development was evaluated in normal mice administered with troglitazone [58]. All nine mice fed with troglitazone developed tumors in the large intestine, in contrast to none of the 10 mice in the control group. An earlier study did not find any tumors in 17 troglitazone-fed normal mice, possibly due to the short duration of feeding (5 weeks in [56] versus 6 months in [58]).

### 4.2. Mammary gland tumors

The mammary gland tumor is another relatively well-studied tumor in animals. Similar to colon carcinogenesis, data on PPAR*γ*'s role in mammary gland carcinogensis suggest a wide range of effect depending on the tumor models (Tables 5 and 6). Some studies indicate no effect, while others suggest that it has a tumor promoting role, while others yet suggest a tumor suppressing role. A murine genetic model supports a tumor-promoting role [60]. In this model, the mammary gland tumor is induced by mammary gland-specific expression of polyoma middle T antigen *(MMTV-PyV)*. Mammary gland specific constitutive expression of PPAR*γ* *(MMTV-VpPPAR*γ*)* did not yield tumor development. However, when crossed with the *MMTV-PyV* mice, the double mutant progeny developed more mammary gland tumors sooner than *MMTV-PyV* mice. The increased tumor burden eventually led to shorter survival. Interestingly, hemizygosity of *PPAR*γ** in the *MMTV-PyV* background did not change the time course of tumor development. Exacerbation of tumor formation by PPAR*γ* was ascribed to increased Wnt-*β* catenin signaling as demonstrated by zebrafish developmental models.

In contrast to this genetic model, chemically induced mammary gland tumors were inhibited by PPAR*γ* agonists. Both TZDs and GW7845, a tyrosine analog, have been shown to exhibit antitumor effects. An early study using nitrosomethylurea (MNU) to induce mammary carcinogenesis showed that GW7845 reduced the incidence, number of tumors *per* animal, and average weight of tumor at autopsy following a two-month administration of the drug to rats [61]. In 7,12-dimethylbenzanthracene (DMBA)-mediated mouse carcinogenesis model, the animals develop multiple types of tumor, including mammary ductal papilloma and adenocarcinoma. Incidence of mammary gland tumor was significantly higher in *Ppar*γ*^+/−^* mice than in *Ppar*γ*^+/+^* mice. The hemizygous mice also had increased number of tumors and a lower survival rate [62].

Spontaneous tumor formation was also examined in *Ppar*γ*^+/−^* mice. Dose reduction of PPAR*γ* does not make animals prone to increased carcinogenesis [62]. In concordance with this finding, the specific deletion of PPAR*γ* in mouse mammary epithelia failed to induce mammary tumors in 20 mice observed for 12 months [63].

### 4.3. Other cancers

In a murine prostate cancer model, generated using tissue-specific SV40 T antigen, reduced *Ppar*γ*^+/−^* had no effects on tumor incidence, latency, size, histopathology, or disease progression [64]. However, in a murine follicular thyroid cancer model containing a dominant-negative mutant form of thyroid hormone receptor **β* (TR*β*^PV/PV^)*, loss of one *PPAR*γ** allele led to increased weight of tumor-bearing thyroid gland, increased lung metastasis, and shortened survival. In addition, rosiglitazone treatment of *TR*β*^PV/PV^* mice reduced thyroid weight, and tumor progression [65], suggesting a tumor-suppressing role for PPAR*γ*. Lastly, in gastric carcinoma, induced with MNU, *PPAR*γ** haploinsufficient mice had increased tumor incidence and shorter survival. Troglitazone treatment significantly reduced tumor incidence in mice with wild-type *PPAR*γ** background [66].

In summary, results from animal studies regarding the role of PPAR*γ* are conflicting and difficult to assess. For the purpose of clarification, we attempted to analyze the published data according to the cancer types, tumor induction models, PPAR*γ* activation/reduction methods, and tumor characteristics (Tables 5 and 6). Our extensive analysis revealed no clear pattern. However, some trends have been noted: (1) in multiple types of carcinogen-induced tumor (Table 5, light grey shaded rows), PPAR*γ* seems to have a tumor-suppressing function. This appears to be independent of how PPAR*γ* is activated or reduced, whereas in genetic tumor models (Table 5, un-shaded rows), the receptor exhibited all possible different effects. As to spontaneous tumors (Table 5, dark grey shaded rows), long-term use of troglitazone increased tumor formation, whereas PPAR*γ* reduction had no effect; (2) a reduction of PPAR*γ* dose by itself (Table 6, light grey shaded rows) is insufficient to induce spontaneous tumor formation, but in existing tumors, it either exacerbates tumor formation or have no effect at all; (3) TZDs (Table 6, un-shaded rows), in most cases, inhibits tumor formation with a rare exception of *Apc^+/Min^* mice.

The activity of the Wnt/*β*-catenin signaling pathway might account for these seemingly discrepant results, as tumor models generated by APC mutation or polyoma middle T antigen all involve overly active Wnt/*β*-catenin signaling. TZDs are shown to induce *β*-catenin in colon [55]. Paradoxically, reduction of PPAR*γ* (Ppar*γ* ^+/−^) also increases *β*-catenin expression in colon [57]. The appropriate activation of PPAR*γ* signaling might also be important. Ligand-independent constitutive activation of PPAR*γ* is involved in the development of mammary gland tumors [60] as well as in the action of PAX8-PPAR*γ* in follicular thyroid carcinoma [29].

## 5. CLINICAL TRIALS OF TZDs IN HUMAN MALIGNANCIES

As discussed above, TZDs have been shown in many preclinical studies to possess antitumor effects that have prompted several early-phase clinical studies to evaluate their efficacies in various types of cancers. In this review, we analyze these studies both in terms of clinical responses and biological responses, focusing on recently published studies that include more than 10 patients (Table 7).

 A phase II clinical trial of rosiglitazone in 12 patients with liposarcoma was recently conducted. Eight of 12 patients were fully evaluated for up to 16 months. As to clinical response, all patients progressed while on treatment with a mean time-to-progression of 5.5 months. Histological appearance of repeated biopsy materials did not show any signs of tumor differentiation. In one of the 8 patients, PPAR*γ* and fatty acid binding protein (FABP) were induced after 12-week rosiglitazone therapy, but disease in this patient progressed similarly to the others [68]. Ten patients with thyroid cancers were treated with rosiglitazone. Among them, 4 had partial response, 2 had stable disease, and the remaining 4 progressed. No correlation was found between the clinical response and levels of PPAR*γ* mRNA and protein in these patients. PAX8-PPAR*γ* status was not assessed [69]. An early study evaluated efficacy of troglitazone in 25 patients with metastatic colorectal carcinoma. All 25 patients progressed with a median time-to-progression of 1.6 months and a median survival time of 3.9 months [70].

In breast cancer, data from two human trials have been published. An early trial on 22 women with refractory breast cancer showed no objective response to troglitazone in 18 of the 21 evaluable patients at 8 weeks after treatment. The therapy was terminated in 16 patients due to progression of their tumors. At 8 weeks, only three patients had stable disease. All patients were evaluated for serum tumor markers, CEA and CA27.29, which showed increased levels within 8 weeks of treatment. Expression of PPAR*γ* was not determined in the study [71]. A short-term pilot trial of rosiglitazone in 38 women with early stage breast cancer was conducted. Clinical response was not assessed in this short-term (<6 week) study. Biological response, as assessed by Ki-67 staining on biopsy tissues before and after treatment, was not detected in treated patients, either. Decreased insulin levels and increased insulin sensitivity were noted in these patients, suggesting that the rosiglitazone did affect metabolism as expected [72].

An early phase II trial of troglitazone in 41 patients with metastatic prostate cancer showed a decrease in levels of prostate-specific antigen (PSA) in 20% of patients enrolled in the study. Prolonged stabilization of PSA was seen in 39% of patients [73]. However, these encouraging results were not reproduced in a large double-blind, randomized, placebo-controlled trial of rosiglitazone in 106 patients with recurrent prostate cancer [74]. The time-to-disease-progression was not significantly different between the rosiglitazone and placebo groups. Moreover, the PSA doubling time, a predictor of clinical recurrence, was also not prolonged by the treatment.

 Taken together, TZDs appear to show little benefit, both in terms of clinical response and biological response, in treating various types of human cancers despite promising results from preclinical animal studies. It is worth noting that most of the studies use low doses of TZDs which are sufficient to activate PPAR*γ* and control diabetes. It remains possible that higher doses, even via receptor-independent pathways, would be beneficial for cancer patients. However, one should keep in mind that TZDs are not a class of drugs without dose-limiting toxicities. Troglitazone was withdrawn from the market by the FDA in 2002 due to liver toxicity. Most recently, increased cardiovascular risk has been associated with rosiglitazone in the diabetic patient population [75, 76] which has prompted the FDA to issue label warnings.

## 6. TZDs AS CHEMOPREVENTIVE AGENTS IN EPIDEMIOLOGY STUDIES

The clinical trials discussed above suggest that TZDs have questionable efficacy as chemotherapeutic agents in patients who already have cancers. Do they have the potential to act as chemopreventive agents? Recently, a large epidemiologic study, involving a population of 87,678 veteran men with diabetes, attempted to answer that question [77]. In this retrospective study, incidence of lung, prostate, and colon cancer in TZD users was compared to incidence in non-TZD users and risk of cancer development was analyzed. Only patients who obtained a cancer diagnosis after the date of TZD initiation were included. TZD usage significantly reduced risk of lung cancer by 33%. It also reduced risk of colon and prostate cancer, though without statistical significance. Interestingly, although the risk of prostate cancer is not significantly influenced by TZDs in the entire population, when examining distinct populations, TZDs are associated with an increased incidence of prostate cancer in both Caucasians and African Americans. These data suggest that the overall reduced risk is accounted for by the non-Caucasian, non-African Americans populations in the study. These data suggest that TZDs may be beneficial for reducing certain cancers in certain populations. Specific molecular abnormalities in specific cancers and the genetic background of different populations may account for these apparently different results.

Although this study was quite strong, we suggest the following for future investigations: (1) separate TZD-users into those using rosiglitazone and those using pioglitazone. In the cardiovascular risk studies, it was shown that rosiglitazone increases the risk while pioglitazone decreases the risk [78]. (2) Evaluate the impact of the duration of TZD exposure on risk of cancer development. (3) Determine the influence of TZDs on the behavior of existing cancers.

## 7. CONCLUSIONS

In this article, we reviewed literature on the roles of PPAR*γ* in cancer with an emphasis on those that suggest a proneoplastic function for the receptor. PPAR*γ*, unlike MYC, RAS, or p53, is neither a strong tumor promoter nor a tumor suppressor. However, it may function as a “conditional tumor promoter” or a “conditional tumor suppressor” that modulates the tumorigenic process depending upon cellular conditions, tumor types, or genetic background of an animal strain or human individuals. TZDs, as a class of pharmacological agent, may have receptor-independent antineoplastic effects, especially at doses higher than diabetic doses or after long-term use and accumulation. It remains possible that their antitumor activities would be enhanced when in combination with other drugs. Further investigation is needed to address that possibility. To help clarify the roles of PPAR*γ* in cancer, future large epidemiological studies of diabetic populations with concurrent cancers would be helpful. In addition, investigations relating PPAR*γ* activities to the clinical outcomes of cancer patients would also be informative.

## Figures and Tables

**Table 1:** EC_50_ of common PPAR*γ* agonists in transactivation assays.

**Agonists**	**Constructs used for transactivation**	**EC_50_ (***μ***M)**	**References**
Ciglitazone	mPPAR*γ*1 LBD^(a)^-GAL4 DBD^(b)^	3	[5]

Pioglitazone	Wild-type mPPAR*γ*1	0.4	[1]
	Wild-type mPPAR*γ*2	0.4	
	mPPAR*γ*1^(c)^ LBD-GAL4 DBD	0.55	[6]
	hPPAR*γ*1^(d)^ LBD-GAL4 DBD	0.58	

Rosiglitazone	Wild-type mPPAR*γ*1	0.03	[1]
	Wild-type mPPAR*γ*2	0.1	
	mPPAR*γ*1 LBD-GAL4 DBD	0.076	[6]
	hPPAR*γ*1 LBD-GAL4 DBD	0.043	

Troglitazone	mPPAR*γ*1 LBD-GAL4 DBD	0.78	[6]
	hPPAR*γ*1 LBD-GAL4 DBD	0.55	

15d-PGJ_2_	Wild-type mPPAR*γ*1	2	[7]
	mPPAR*γ*1 LBD-GAL4 DBD		

(a) LBD, ligand binding domain. (b) DBD, DNA binding domain. (c) mPPAR*γ*1, mouse PPAR*γ*1. (d) hPPAR*γ*1, human PPAR*γ*1.

**Table 2:** Peak plasma concentrations of PPAR*γ* agonists.

**Agonists**	**C**_max_^(a)^ (**μ**M)	**References**
Ciglitazone	15~30^(b)^	[8]
Pioglitazone	0.2~2.5	[9]
Rosiglitazone	0.2~1.7	*Avandia* Prescribing Information^(c)^
Troglitazone	0.7~8.8	[10]
15d-PGJ_2_	Low nanomolar to picomolar range^(d)^	[11]
		[12]

(a)*C*_max_, the maximum or peak plasma concentration in human unless otherwise indicated. (b)That in dog plasma. (c)From http://us.gsk.com/products/assets/us_avandia.pdf. (d)Physiological concentrations in cerebrospinal fluid, urine, and the interior of adipocytes.

**Table 3:** Points to be considered to discern drugs/TZDs versus receptor effects.

(1) Are high or low doses of drugs used in the studies with respect to their *K_d_* values for PPAR*γ*, or plasma concentrations?
(2) Are multiple pharmacological agents of different chemical classes used?
(3) Are any antagonists included in the study?
(4) Are any genetic approaches used to confirm the pharmacological findings?

**Table 4:** PPAR*γ* expression in human tumor versus normal tissues.

**Tumor versus normal tissue**	**No. of cases**	**References**
***Overexpression***		
Prostate cancer/prostatic intraepithelial neoplasia	156/15	[13]
Renal cell carcinoma	126	[14]
Nonsmall-cell lung carcinoma	22	[15]
Hepatocellular carcinoma/lymph node metastasis	20/6	[16]
Squamous cell carcinoma	20	[17]
Metastatic breast adenocarcinoma	6	[20]
Infiltrating ductal breast adenocarcinoma	3	[18]
Papillary thyroid carcinoma	6^(a)^	[19]
***Increased expression during tumor progression***		
Breast adenocarcinoma	1^(b)^	[20]
Ovarian carcinoma	28 versus 28^(c)^	[21]
Urinary bladder carcinoma	100 versus 70^(d)^	[22]
Pancreatic ductal adenocarcinoma	45 versus 84^(e)^	[23]
***Similar expression***		
Colorectal adenocarcinoma	11	[24]
Gastric adenocarcinoma	12	[25]
Liposarcoma	13	[26]
Adrenocortical tumors	32	[27]

(a) Of the six papillary carcinoma tissues, three expressed PPAR*γ* mRNA. (b) The primary and metastatic breast cancer cell lines were derived from a single patient. (c) Normal, benign, or borderline versus malignant tumors (grades 1, 2, and 3). (d) Lower (≤pT1) versus higher (≥pT2) tumor stages. (e) Lower (pT1 & pT2) versus higher (pT3 & pT4) tumor stages.

**Table 5:**
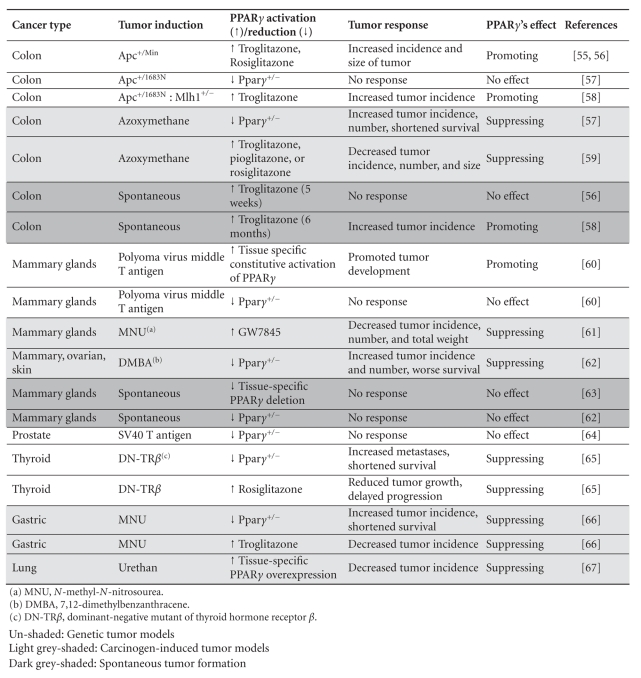
PPAR*γ* and agonists in animal models (differentially shaded according to methods of tumor induction).

**Table 6:**
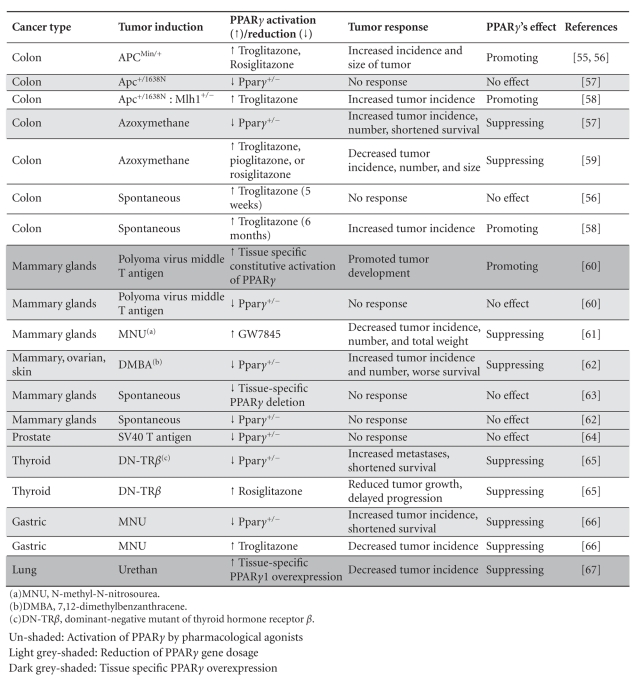
PPAR*γ* and agonists in animal models (differentially shaded according to methods of PPAR*γ* manipulation).

**Table 7:** Clinical trials of TZDs in cancer patients.

**Cancer type**	**Phase**	**TZDs**	**No. of pts**	**Tumor response**	**References**
Liposarcoma	II	Rosiglitazone	12	All patients progressed, no sign of differentiation by histology	[68]
Thyroid cancer	I, II	Rosiglitazone	10	4 pts with partial response, 2 with stable disease, and 4 with progressed disease	[69]
Metastatic colorectal cancer	I, II	Troglitazone	25	All patients progressed	[70]
Refractory breast cancer	II	Troglitazone	22	Most patients progressed with increased serum tumor markers	[71]
Early-stage breast cancer	II	Rosiglitazone	38	No reduction in Ki-67 staining on tissue biopsies	[72]
Metastatic prostate cancer	II	Troglitazone	41	Decrease or stabilization of PSA	[73]
Recurrent prostate cancer	III	Rosiglitazone	106	Similar to placebo in both PSADT and time-to-disease-progression	[74]
